# Complete mitochondrial genome sequence of the Jayakar’s seahorse *Hippocampus jayakari* Boulenger, 1900 (Gasterosteiformes: Syngnathidae)

**DOI:** 10.1080/23802359.2017.1372704

**Published:** 2017-09-01

**Authors:** Rubin Cheng, Yun Fang, Yuqing Ge, Qiang Liu, Guangji Zhang

**Affiliations:** a College of Pharmaceutical Science, Zhejiang Chinese Medical University, Hangzhou, P. R. China;; b The First Affiliated Hospital, Zhejiang Chinese Medical University, Hangzhou, P. R. China

**Keywords:** *Hippocampus jayakari*, Genus *Hippocampus*, mitochondrial genome, evolutionary relationship

## Abstract

In the present study, we report the complete mitochondrial genome of Jayakar’s seahorse, *Hippocampus jayakari* Boulenger, 1900. The genome of *H. jayakari* is found to be 16,520 bp in length and has a base composition of A (32.82%), C (23.28%), G (14.13%), and T (29.67%). Similar to other Hippocampus species, it contains a typically conserved structure including 13 protein-coding genes, 2 rRNA genes, 1 control region (D-loop), and 22 tRNA genes. The protein-coding genes had ATG or GTG as the initiation codon and only 6 genes (COX1, ATP8, ATP6, ND4L, ND5 and ND6) terminated by the complete typical stop codon TAA. The lengths of 12S rRNA and 16S rRNA are 939 bp and 1686 bp, respectively. All tRNA genes typically formed a cloverleaf secondary structure, except for tRNA-Ser containing a dihydrouridine arm replacement loop. *Hippocampus jayakari* exhibited a relatively distant genetic relationship with other 13 Hippocamupus species according to the phylogenetic analysis. The complete mitochondrial genome sequence provided here would be useful for further understanding the evolution of *Hippocamupus* and conservation genetics of *H. jayakari*.

The Jayakar’s seahorse, *Hippocampus jayakari* Boulenger, 1900 (Gasterosteiformes: Syngnathidae) is one of the spiny seahorse with spines on alternate tail rings and mainly distributed in the Western Indian Ocean, Red Sea and Arabian Sea (Lourie et al. 2004). *Hippocampus Jayakari* is a demersal seahorse, with a maximum reported depth of 20 m, and often found in rubble-algae with sparse seagrass, soft-bottom on sponges and seagrass beds (Kuiter 2000). Since the overexploitation and habitat destruction, this species is also listed in Appendix II of CITES and as Data Deficient by the IUCN Red List of Threatened Species. Although the accurate size of trade in *H. Jayakari* is unknown, it is an important adulteration of *Hippocampus* in traditional Chinese medicine market (Wen et al. 2013).

Here, we sequenced and characterized the complete mt genome of *Hippocampus jayakari*. The specimen of *H. Jayakari* was purchased from Chinese materia medica market in Anguo city of Hebei Province and identified based on its morphometric and meristic characteristics, such as the double cheek spine, spines with a broad dark band near tip and the dark midventral line (Lourie et al. 2004). The seahorse sample of *H. Jayakari* (JS31-01) was deposited in the collection centre of College of Pharmaceutical Science at Zhejiang Chinese Medical University. Samples used in this study were with Animal Ethics approval for experimentation granted by Zhejiang Chinese Medical University. Total genomic DNA was extracted from the muscle tissue from tail using a Tiangen DNA extract kit (Tiangen Inc., Beijing, China) following the manufacturer’s instructions. The complete mtDNA of *H. Jayakari* was amplified and sequenced by 14 pairs of primers designed according to the published mitochondrial genome sequences in the genus *Hippocampus* (Song and Mabuchi 2014; Cheng et al. 2017; Wang et al. 2017). The mt genome sequence of *H. Jayakari* with the annotated genes was deposited in GenBank under the accession number of KX890469. A total of 17 complete mt genomes in family Syngnathidae were collected and the phylogenetic relationships were inferred utilizing maximum-likelihood (ML) methods by MEGA 7.0 based on the concatenated supergene consisting of 13 mitochondrial protein-coding genes (Kumar et al. 2016).

Whole mitochondrial genome sequence of *H. Jayakari* has a circular genome of 16,520 bp, containing 13 protein-coding genes, 2 rRNA genes, 1 control region, and 22 tRNA genes. The contents of A, C, G, and T are 32.82%, 23.28%, 14.13%, and 29.67%, respectively. AT and GC contents of mt genome are 62.49% and 37.51%, respectively. The proportion of coding sequences with a total length of 11,129 bp is 67.37%, which encodes 3798 amino acids. All protein-coding genes in *H. Jayakari* started with a typical ATG codon, except for COX1 that was initiated by a GTG start codon. For the stop codon, only COX1, ATP8, ATP6, ND4L, ND5 and ND6 genes ended with complete TAA, the other seven genes terminated with a single base T or TA. Incomplete stop codon was found in the mitochondrial genes of many other fish species (Yu and Kwak 2015; Huang et al. 2017). The lengths of 12S ribosomal RNA and 16S ribosomal RNA are 939 bp and 1686 bp, respectively. The 22 tRNA genes vary from 66 to 77 bp in length. The tRNA-Ser gene contains a dihydrouridine arm replacement loop and the other 21 tRNA genes could be folded into the typical cloverleaf secondary structure. The control region located between tRNA-Pro and tRNA-Phe gene was 879 bp in length, ranging from 15,642 to 16,520 bp.

Phylogenetic relationships among *H. Jayakari* and other 17 species in Syngnathidae with complete mitogenome sequences available on GenBank were constructed using pipefish species as outgroup. As shown in Figure 1, the *H. Jayakari* forms a monophyletic group with other seahorse species with a high bootstrap support value, indicating relatively distant genetic relationships with the currently sequenced Hippocampus species. The data of *H. Jayakari* could serve to enrich the resource of seahorse in systematic, population genetic, and evolutionary biological studies.

**Figure 1.**
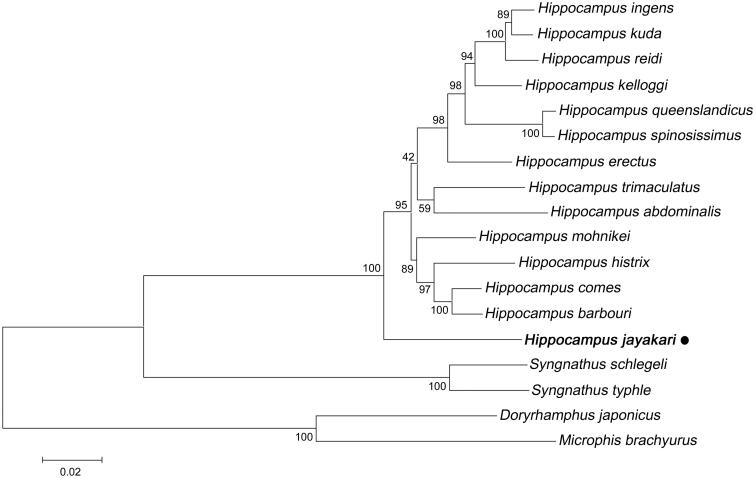
Maximum-likelihood (ML) phylogenetic tree of *H. jayakari* and the other 17 species in Syngnathidae using 2 Syngnathus species, *Doryrhamphus japonicus* and *Microphis brachyurus* as outgroups. Number above each node indicates the ML bootstrap support values generated from 100 replicates. All 17 species’ accession numbers are listed as below: *Hippocampus ingens* NC_024530, *H. kuda* NC_010272, *H. reidi* NC_027931, *H. kelloggi* NC_029349, *H. queenslandicus* NC_034319, *H. spinosissimus* NC_029350, *H. erectus* NC_022722, *H. trimaculatus* NC_021107, *H. abdominalis N*C_028181, *H. mohnikei* NC_030251, *H. histrix* NC_021454, *H. comes* NC_020336, *H. barbouri* NC_024536, *Syngnathus schlegeli* AP012318, *Syngnathus typhle* NC_030279, *Doryrhamphus japonicus* NC_024187, *Microphis brachyurus* NC_010273.
